# Planetary Energy Flow and Entropy Production Rate by Earth from 2002 to 2023

**DOI:** 10.3390/e26050350

**Published:** 2024-04-23

**Authors:** Elijah Thimsen

**Affiliations:** Department of Energy, Environmental and Chemical Engineering, Washington University in Saint Louis, Saint Louis, MO 63130, USA; elijah.thimsen@wustl.edu; Tel.: +1-314-935-6103

**Keywords:** nonequilibrium thermodynamics, life, ecosystems, evolution, entropy generation, geoengineering, social sciences, albedo, externalities

## Abstract

In this work, satellite data from the Clouds and Earth’s Radiant Energy System (CERES) and Moderate Resolution Imaging Spectroradiometer (MODIS) instruments are analyzed to determine how the global absorbed sunlight and global entropy production rates have changed from 2002 to 2023. The data is used to test hypotheses derived from the Maximum Power Principle (MPP) and Maximum Entropy Production Principle (MEP) about the evolution of Earth’s surface and atmosphere. The results indicate that both the rate of absorbed sunlight and global entropy production have increased over the last 20 years, which is consistent with the predictions of both hypotheses. Given the acceptance of the MPP or MEP, some peripheral extensions and nuances are discussed.

## 1. Introduction

The idea that the Earth is regulated by life for life rings true. Beyond the impact of humans on the environment [1], early prehistoric photosynthetic organisms are believed to have changed the composition of the atmosphere to be oxygen rich [2]. Since then, global photosynthesis has maintained the chemical composition of the atmosphere far away from the local equilibrium state at the surface temperature and pressure of the Earth [3]. Even more remarkable is that this nonequilibrium chemical composition is relatively stable over time periods much longer than the average CO_2_ residence time in the atmosphere [4], which is on the order hundreds of years [5]. Such behavior resulting in a relatively stable, albeit unexpected, chemical state suggests amenability to thermodynamic analysis. The penultimate goal of such an analysis would be to understand what controls the stationary state(s) towards which the Earth is trending, and thereby make probabilistic forecasts.

Principles have been proposed that govern systems which operate over long periods of time and are very far from local equilibrium, such as life itself. Building on phenomenological observations of ecosystems, the Maximum Power Principle (MPP) is an often-cited example of a principle that governs evolution of complex communities containing living organisms. The development of the MPP has been lucidly described in a recent work by Hall and McWhirter [6], and so it will not be repeated here. In brief, the basic idea is that ecosystems evolve, subject to external constraints (e.g., element availability, energy source, etc.), towards stationary states that maximize the flow of energy through the ecosystem per unit time, which is power. There is a related idea that was popularized most recently in a series of papers by Kleidon [7,8,9,10,11,12] and others [13,14,15,16,17] that is termed the Maximum Entropy Production Principle (MEP). The MEP hypothesis basically states that the ecosystem will evolve towards a state where it generates the most entropy per unit time from an external energy flow.

The MPP and MEP hypotheses are related. For example, consider the experimental system recently described by Kondepudi et al. [18,19,20]. In this experiment, a metal pin is placed above an oil bath, and a constant voltage is applied to the pin that is sufficient to ionize air. There is a coaxial metal ring placed into the oil bath that is partially submerged and is also exposed to the air, which acts as a ground electrode that collects charges. Importantly, the oil also contains small metal spheres that are free to move about. Once energized, there is a current of charge via ions generated in the air from the high voltage pin to the grounded ring. Interestingly, the metal spheres in the oil self-assemble into structures that increase the current flow between the pin and the ground electrode. Since the voltage applied to the pin is constant, this increase in current, due to the self-assembly of the metal spheres in the oil, increases the energy flow through the system, which can be thought of as a conversion rate of electrical energy into heat. Thus, this dissipative structure, which includes the mobile metal beads, evolves towards states that maximize the flow of energy through the system, consistent with MPP. Since electrical energy input is work, which does not carry entropy, and the heat generated by the dissipation is at a constant temperature, the entropy production rate also increases because of the self-assembly of the spheres. Thus, the dynamics of the system are consistent with both the expectation of the MPP, which predicts evolution towards states of higher energy flow, and the MEP, which predicts evolution towards states of higher entropy production rate. While this example is clear and helpful for illustrating the principles, the predictions of the MPP and MEP are the same, and so one cannot discriminate between the hypotheses using that system. 

The goal of this work is to test, or at least evaluate, the MPP and MEP hypotheses as they apply to the recent evolution of the Earth and life contained therein, which can also be thought of as a dissipative structure. The first step of a thermodynamic analysis is defining the system boundary and the exchanges of matter, energy, and entropy across that boundary. It is relatively straightforward to draw a boundary around an ecosystem, but describing the exchanges of matter, energy and entropy across that boundary is exceedingly difficult for most subsystems on Earth. However, the situation can be simplified if the entire Earth is taken as the ecosystem. The Earth is nominally a closed system. The matter that crosses the top of the atmosphere [21,22] is negligible compared to the amount of matter contained within the system. Furthermore, the exchanges of energy and entropy across the boundary are dominated by several orders of magnitude by radiation [9]. There are only three main external exchanges between the Earth and outer space: (1) shortwave (SW) radiation input from the sun in the visible and near-infrared regions of the electromagnetic spectrum; (2) SW radiation output that is sunlight scattered from the surface and atmosphere (e.g., clouds) in the visible and near infrared regions of the spectrum; and (3) longwave (LW) thermal radiation in the infrared with a spectrum determined by the temperature of the Earth surface and clouds being in the range of 210 to 325 K. SW corresponds to the wavelength range from 0.2 to 5 μm and LW corresponds to the wavelength range from 5 to 50 μm. When time-averaged and integrated over the entire boundary enclosing the Earth, these radiation exchanges are on the order 10^4^–10^5^ terawatts (TW), several orders of magnitude higher than the next closest energy exchanges. Geothermal heat via volcanic activity from the Earth’s core and radioactive decay, as well as global combustion by human fossil energy utilization, only amount to approximately 10^1^–10^2^ TW on average [9]. Since the other boundary interactions are insignificant, the system is adequately described by the three radiation interactions, and thereby energy flow through the Earth and the entropy production rate by the Earth can be quantified.

Both the MPP and MEP principles predict evolution towards an extremum. More specifically, a stationary state whereat the energy flow through the Earth is maximized in the case of MPP, or entropy production rate by the Earth is maximized in the case of MEP. Energy flow through the Earth is taken to be the amount of absorbed sunlight, since the Earth is approximately, but not precisely, in a steady state and the net energy absorbed at SW wavelengths is balanced by energy outflow at LW wavelengths. The expectation for both the MPP and MEP is that the corresponding variable (energy flow or entropy production rate) will either increase in time (heading towards a maximum) or remain constant (already at a maximum). Since the temperature of the Earth is changing due to global warming, and the albedo of the Earth has changed over the last several decades [23], analysis of recent global trends as characterized by satellite measurements may allow for discrimination between the MPP and MEP hypotheses. 

In this work, recently reported satellite measurements of the power density of (1) incoming SW solar radiation, (2) outgoing SW radiation, and (3) outgoing LW radiation from Earth were combined with satellite measurements of (4) cloud cover area fraction, (5) cloud temperature, (6) sea temperature, and (7) day/night land temperatures to estimate how the absorbed sunlight and net global entropy exchange with space has changed from 2002 to 2023. The key findings are that (1) the rate of sunlight absorption has increased from 2002 to 2023, corroborating recent reports that the albedo has decreased [23]; and (2) the entropy production rate has also increased. In other words, rising global surface temperature from the greenhouse effect has been more than compensated for by the rising rate of sunlight absorption and accumulation of heat in the oceans. The results support both the MPP and MEP hypotheses. The conclusion is that observations made of global temperature distributions and energy fluxes over the last two decades are consistent with both the MPP and MEP hypotheses governing the global ecosystem, including human society. While we cannot rule out either hypothesis completely, it is a notable result that recent satellite measurements are consistent with these hypotheses being true.

## 2. Materials and Methods

**Figure 1:**
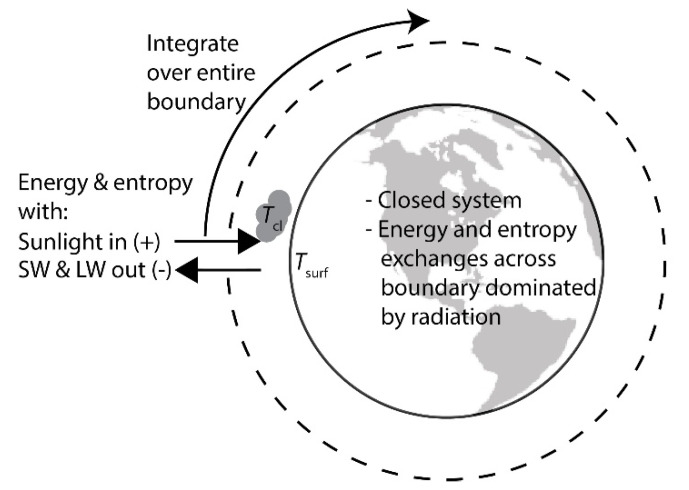
System definition for thermodynamic analysis. Boundary is top of atmosphere.

The analysis provided in this work is based on the boundary interactions at the top of the Earth’s atmosphere. The entire surface of the boundary is fully accounted for on a 1° × 1° grid in longitude and latitude. Internal dynamics of the Earth (such as heat transfer and fluid flow around the planet) are only important insofar as they affect the boundary interactions. For example, if heat is transported from the equator to the pole, and then radiated into space at the pole, it would manifest as a lower LW heat output at the equator and a higher LW heat output at the pole. Since the important interactions are known everywhere on the boundary that encloses Earth, the internal dynamics that produce those boundary interactions are not important because the boundary interactions themselves are known everywhere on the surface (Figure 1).

This research was an analysis of satellite data that has been publicly reported by the United States National Aeronautics and Space Administration (NASA). The period studied was from July 2002 to August 2023. The data was spaced monthly on a 1° × 1° grid in longitude and latitude. Cloud area fraction, cloud temperature [24], SW light input, SW light output, and LW light output were all downloaded as a Top of Atmosphere Energy Balanced and Filled (EBAF-TOA) data product from the Clouds and the Earth’s Radiant Energy System (CERES) instrument [25,26]. SW is the wavelength range from 0.2 to 5 μm and LW is the wavelength range from 5 to 50 μm [25]. Day/night land temperatures [27] and sea temperatures [28] were downloaded from the Moderate Resolution Imaging Spectroradiometer (MODIS) datasets. The data was imported using Origin 2019 Pro (OriginLab, North Hampton, MA, USA) and exported to Matlab R2015a (MathWorks, Natick, MA, USA) for analysis. Contour plots were made in Origin 2019 Pro. 

Data from the MODIS instrument for the day land temperature, night land temperature, and sea temperature were combined to make an effective surface temperature as a function of position (longitude, latitude, and month). If the coordinate was surface water, then the sea temperature was used. If the coordinate was land, then the temperature was time-averaged using the number of daylight hours. In other words, the average land temperature was taken as:

(1) $$T_{land}=xT_{day}+(1-x)T_{night}$$

where *x* is the average number of daylight hours for the location and month divided by the number of hours in the day. The day and night temperatures have the corresponding subscripts. There were a few holes in the MODIS dataset where no temperatures were reported. Since the surface temperature depends primarily on latitude and month, those data holes were filled with the latitudinal average surface temperature for the corresponding month.

Data from CERES was used without modification. For a given pixel, the radiation exchanges were calculated by multiplying a given energy flux by the corresponding area of the pixel:

(2) $$I_{i}(\lambda ,\phi )=q_{i}(\lambda ,\phi )dA={\left(\frac{\pi r}{180{}^{\circ}}\right)}^{2}q_{i}(\lambda ,\phi )\cos(\phi )d\phi d\lambda$$

Here, *i* is the index of the corresponding energy flux (*q*_i_), which depends on position and time (net flux, SW input, SW output, or LW output in units of W m^−2^). The usual definition of longitude (*λ*) and latitude (*ϕ*) has been used and the units of those angles are degrees. The radius of the Earth (*r*) is taken as the average of the polar and equatorial radii (6.367 × 10^6^ m). Since the data is on a 1° × 1° grid, both *dϕ* and *dλ* are taken as 1°.

The system definition with the corresponding boundary interactions is presented in Figure 1. At each location on Earth, there are energy and entropy exchanges with the solar input, SW output, and LW output. These exchanges are integrated over the entire surface of the boundary to get the total exchange. It has been pointed out recently by Gibbins and Haigh that accounting for the accumulation of entropy in the Earth is important for resolving trends in the global entropy production rate [29]. Accumulation of energy and entropy in the system is therefore accounted for. The energy accumulation is calculated from the energy balance and the three boundary interactions. It has been reported that over 90% of the energy accumulated on Earth goes to the heating of the oceans [30], which is consistent with the observed sea surface temperature change over the last 20 years (vide infra). Therefore, it is assumed that the entropy accumulation associated with this energy accumulation is also stored in the oceans.

The energy balance requires integrating the local energy fluxes over the entire boundary at the top of the atmosphere. For a given position as defined by a longitude–latitude pair, the net energy exchange can be written using Equation (2):

(3) $$I_{net}(\lambda ,\phi )=q_{net}(\lambda ,\phi )dA={\left(\frac{\pi r}{180{}^{\circ}}\right)}^{2}\left(q_{sun,in}-q_{SW,out}-q_{LW,out}\right)\cos(\phi )d\phi d\lambda$$

Here, the sunlight input, SW output, and LW output are all provided explicitly by the CERES data product as a function of position and time, and the sign convention is positive going in and negative going out (Figure 1). The total energy exchange with space can be calculated by integrating Equation (3) over the entire boundary:

(4) $$Q_{net}=\int_{earth}q_{net}(\lambda ,\phi )dA={\left(\frac{\pi r}{180{}^{\circ}}\right)}^{2}\int_{-180{}^{\circ}}^{180{}^{\circ}}\int_{-90{}^{\circ}}^{90{}^{\circ}}\left(q_{sun,in}-q_{SW,out}-q_{LW,out}\right)\cos(\phi )d\phi d\lambda$$

where *Q*_net_ has units of power (e.g., terawatt, TW). Equation (4) and other equations of similar form were integrated numerically. Since all three energy fluxes are provided in the CERES data set, Equation (4) can be integrated without any assumptions. A non-steady energy balance can then be written assuming this energy is accumulated as internal energy:

(5) $${\dot{U}}_{p}=Q_{net}$$

where ${\dot{U}}_{p}$ is the change in the internal energy of the planet with time.

Similar to the energy balance, the entropy balance requires integrating the fluxes over the entire boundary at the top of the atmosphere. There is an entropy exchange with space for each of the energy exchange terms, and so we can write the net entropy exchange in the same way as the energy exchange:

(6) $$S_{e}=\int_{earth}s_{e}(\lambda ,\phi )dA={\left(\frac{\pi r}{180{}^{\circ}}\right)}^{2}\int_{-180{}^{\circ}}^{180{}^{\circ}}\int_{-90{}^{\circ}}^{90{}^{\circ}}\left(s_{sun,in}-s_{SW,out}-s_{LW,out}\right)\cos(\phi )d\phi d\lambda$$

In Equation (6), the terms in the bracket correspond to the entropy flux due to the solar input (*s*_sun,in_), reflected SW output (*s*_SW,out_), and emitted LW thermal radiation output (*s*_LW,out_), all of which depend on position and time. To be clear, for the Earth system, most of the entropy produced by the system is flushed out by the outgoing LW radiation, which can be seen in Equation (6). The global entropy production rate (GEP) can be calculated from an unsteady entropy balance:

(7) $${\dot{S}}_{P}=S_{e}+GEP$$

where ${\dot{S}}_{P}$ is the entropy accumulation in the planet, which is assumed to be primarily in the oceans. Since this entropy accumulation is primarily through the heating of the oceans, we estimate a lower limit for the planetary entropy accumulation as ${\dot{S}}_{P}\approx {\dot{U}}_{P}/T_{sea}$, where both the planetary internal energy accumulation ${\dot{U}}_{P}$ (Equation (5)) and area-weighted average sea surface temperature $T_{sea}$ change with time.

The task now becomes to describe the entropy fluxes from quantities that are known from the MODIS and CERES datasets. Energy fluxes are proportional to entropy fluxes by an effective temperature (*T*_i_):

(8) $$q_{i}(\lambda ,\phi )=T_{i}s_{i}(\lambda ,\phi )$$

All of the energy fluxes are known from the CERES dataset, and thus only the corresponding effective temperatures are left to be described as a function of space and time.

The easiest of the temperatures is the solar input. Radiation is emitted from the sun with a temperature of approximately 5700 K. As it travels through space, the spectral distribution remains mostly unchanged and similar to a blackbody at the surface temperature of the sun. However, the energy flux through a projection of constant area, such as a disk, decreases with distance from the sun; which causes the spectral temperature of the sunlight (*T*_sun,in_ in Equation (8)) to decrease. Expressions that relate entropy flux to the spectral irradiance of a nonequilibrium light source have been known for decades [31,32,33]. The basic idea is that for each wavelength bin, one can find a blackbody temperature that would produce the irradiance at that wavelength, and then calculate the corresponding entropy flux. By summarizing the entropy flux contributions of each wavelength bin, the total entropy flux can be calculated for an arbitrary nonequilibrium irradiance spectrum. Performing such a calculation for the air mass zero (AM0) spectrum that describes the sunlight incident upon the top of the atmosphere, the spectral temperature of the solar input can be estimated as approximately *T*_sun,in_ = 1200 K, which is notably lower than the surface temperature of the sun.

The dependence of the spectral temperature on energy flux is relatively weak. For example, the irradiance at the surface of the sun estimated using the Stephan–Boltzmann law is approximately 10,000 times higher than at the top of Earth’s atmosphere, and yet the spectral temperature of sunlight at the surface of the sun is only 4.6 times higher. Since the reflected SW light from the Earth has an irradiance approximately 30% of the incident SW sunlight, and the dependence of the spectral temperature on irradiance is relatively weak, it is reasonable to assume that:

(9) $$T_{sun,in}=1200K\approx T_{SW,out}$$

in the absence of any detailed spectral irradiance data for the SW output. Stephens and O’Brian have calculated the entropy and energy fluxes for several different types of SW scattering, specifically from tropical, mid-latitude and subarctic surfaces, as well as cirrus and cumulus clouds [34]. Using their data, and dividing the energy flux by the entropy flux (Equation (8)), the scattered SW radiation from all of these objects has a spectral temperature of 1195 ± 82 K, in excellent agreement with Equation (9) (see Stephens and O’Brian [34] Table 1). It is worth noting that if the spectral irradiance of the SW output were known (for example by more sophisticated broadband hyperspectral satellite measurements in the future) then spectral temperature of the SW energy outflow *T*_SW,out_ or the outgoing entropy flux with SW radiation, *s*_SW,out_, could be calculated directly as a function of position and time.

The final effective temperature is associated with the LW thermal emission, *T*_LW,out_, which is expected to be in the range of surface and cloud temperatures. Again, it is emphasized that if the spectral irradiance of the LW radiation output were known, the entropy flux *s*_LW,out_ could be explicitly calculated without the need for any approximations involving temperatures reported by the CERES and MODIS instruments. Unfortunately, the spectral irradiance of the radiation outputs is not currently known with sufficient spectral and spatial resolution, and thus simplifying approximations must be made to use the available data. For each pixel, surface and cloud temperatures are known. Additionally, the CERES data set includes the fraction of the pixel area that is estimated to be covered by clouds [24]. The entropy flux with the LW output from a pixel is calculated by assuming that the LW energy flux is partitioned between the cloud and surface according to the cloud area fraction:

(10) $$s_{LW,out}(\lambda ,\phi )=\frac{4}{3}q_{LW,out}\left(\frac{f_{cloud}}{T_{cloud}}+\frac{1-f_{cloud}}{T_{surf}}\right)$$

where *f*_cloud_ is the fraction of the pixel area that is cloud covered, *T*_cloud_ is the cloud temperature reported in the CERES dataset, and *T*_surf_ is the sea temperature if the pixel is water, or the land temperature calculated using Equation (1) if the pixel is land. The factor of 4/3 in Equation (10) comes from the fact that photons carry momentum and exert pressure, meaning radiation boundary interactions are more complicated than pure heat transfer. For further information on this point, see the detailed work of Wu and Liu [35], who present a derivation. To test the effect of the factor 4/3 in Equation (10), calculations were performed by omitting it as well, and all the qualitative conclusions about how the global entropy production rate changed with time were the same in that case where the factor of 4/3 was replaced by a factor of 1. The spectral approach that was used to calculate the effective temperature of the solar input and SW output (Equation (9)) accounts for the ability of photons to exert pressure, which makes inclusion of the factor 4/3 in Equation (10) consistent with Equation (9).

**Figure 2:**
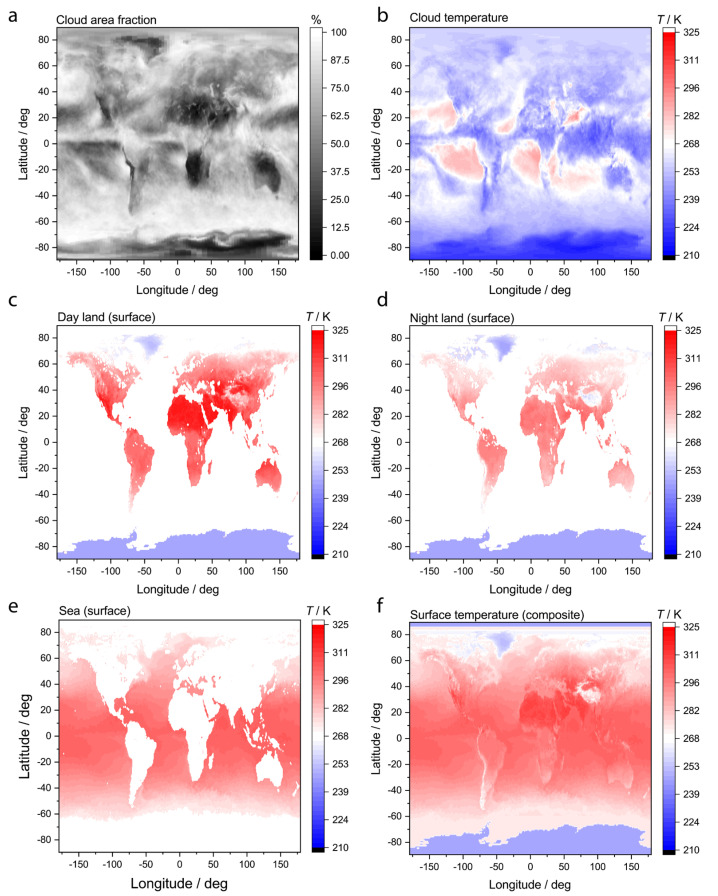
Example snapshot of temperature data averaged in May 2005. (**a**) Cloud area fraction, (**b**) cloud temperature, (**c**) land surface temperature during daytime, (**d**) land surface temperature during nighttime, (**e**) surface temperature of sea, and (**f**) composite surface temperature of land and sea using time-average of day and night land temperatures.

An example of a complete set of temperature data required to calculate the total entropy exchanged with space using Equation (6) for one time point is provided in Figure 2. The cloud area fraction is presented in Figure 2a, the cloud temperature in 2b, and the composite surface temperature in Figure 2f with the constituents used to construct that composite in Figure 2c–e.

The Earth has an annual cycle. The planet does not follow a perfect circular orbit with the sun at the middle. In addition to the summer/winter cycle, the Earth is closest to the sun during the Southern Hemisphere summer, which results in a higher global solar input during that time of year. The annual cycle causes a periodic trend in all the global radiation exchanges with space that has a period of one year (vide infra). The magnitude of the annual variation is rather large. For example, consider the swing in temperature from summer to winter compared to the magnitude of the temperature change due to global warming. To reveal trends in the underlying energy and entropy fluxes and associated surface temperatures, it is helpful to compensate for the annual cycle. In this work, the annual cycle is compensated for by considering the value of a variable in a given month (energy flux, entropy flux, temperature, etc.) relative to the value of that variable in the same month of a reference year. More specifically, consider the value of some variable *K*_i_ that varies with time and longitude/latitude:

(11) $${\bar{K}}_{i}^{j,z}=K_{i}^{j,z}-K_{i}^{j,ref}$$

where the overbar denotes a relative value. The superscripts denote that it is a value for month *j* of year *z*. The superscript *ref* denotes the reference year. While the year *z* may increment through time, the reference year *ref* is a constant. The reference year chosen for this work is 2003, which is the earliest year for which a complete dataset was available.

## 3. Results

**Figure 3:**
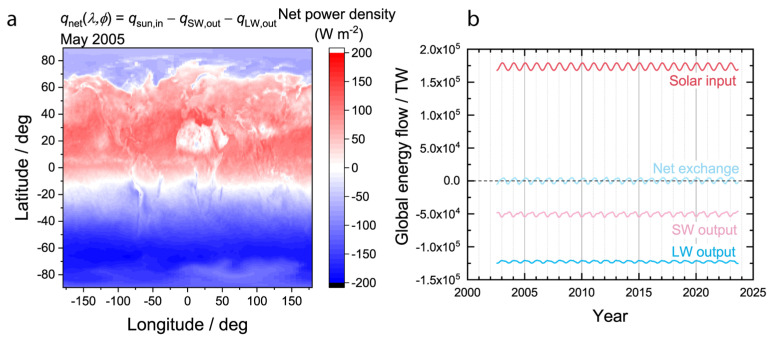
Net exchanges of radiation with space. (**a**) Example map of net radiation exchange as a function of position for May 2005. (**b**) Globally integrated radiation exchanges as a function of time.

Since the LW thermal emission is isotropic but the SW exchanges are not, the net exchange of radiation with space is negative (outgoing) in locations and months of the year where the incoming solar radiation is weak, such as the winter, and positive (incoming) at locations and months of the year where the sunlight is strong, such as the summer. To illustrate this important trend, an example map of the net radiation exchange for an arbitrary month (May 2005) is plotted in Figure 3a. From approximately −10° to 60° latitude there was a net absorption of energy, while south and north of this band there was a net emission of energy into space. The band of net radiation absorption and bands of net radiation emission shift throughout the year with the annual cycle.

There is very little accumulation of energy in the Earth over time when compared to the magnitude of the radiation exchanges. Plotted in Figure 3b are the globally integrated solar input, SW output, and LW output as a function of time from 2002 to 2023. The solar input displays an annual cycle according to the distance of the planet from the sun. The net exchange, calculated by summing all three radiation contributions at a given time point via Equation (4), oscillates about zero with a very small amplitude and a period of one year. By compensating for the small variations of the annual cycle using relative values via the approach outlined in Equation (11), effects of the tiny oscillations about zero with a period of one year can be removed to reveal long term trends.

**Figure 4:**
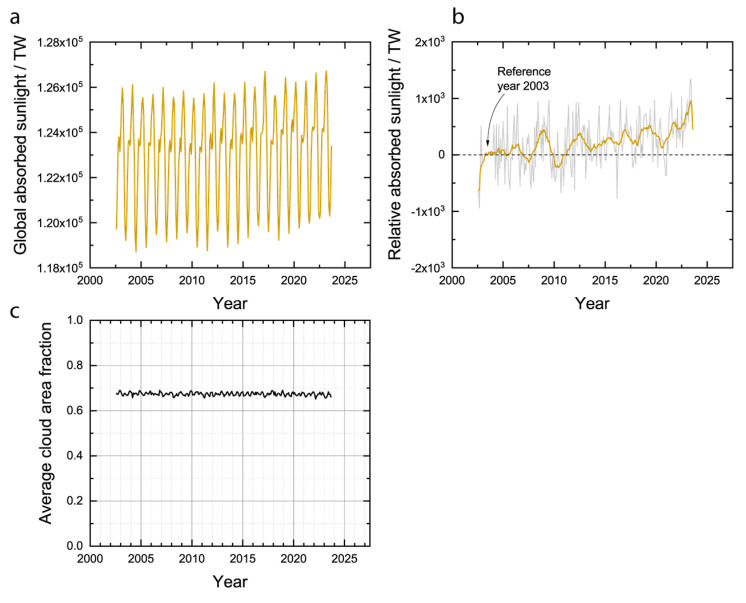
Absorbed sunlight. (**a**) Globally integrated solar input minus SW output. (**b**) Globally integrated solar input minus SW output relative to 2003. Grey line is raw data in (**b**) and orange line is moving average with 12-month window. (**c**) Area-weighted average cloud area fraction calculated from CERES dataset.

Applied to the entire planetary ecosystem, the expectation of the MPP is that evolution should occur in such a way that sunlight absorbed by the Earth will increase (approaching a maximum) or stay the same (already at the maximum). Assuming a steady state, the amount of sunlight absorbed by the Earth can be calculated as the globally integrated solar input minus the SW output. The globally absorbed sunlight, so calculated, is plotted in Figure 4a without removing the effect of the annual cycle and in Figure 4b as a relative value calculated using Equation (11). Even with data that is uncompensated for the annual cycle (Figure 4a), a trend of increasing maxima and minima can be seen that already shows that the rate at which the Earth is absorbing sunlight appears to have increased over the last 20 years. Plotting the same data relative to the year 2003 (Figure 4b), a moving average with a window of 12 months more clearly shows the magnitude by which the amount of absorbed sunlight has increased over the last 20 years—approximately 1000 TW. This trend is consistent with the expectation of the MPP. Furthermore, the area-averaged fraction of cloud cover has decreased slightly over the last 20 years, from approximately 68% to 67% (Figure 4c), which could explain a portion of the increased absorbed sunlight.

**Figure 5:**
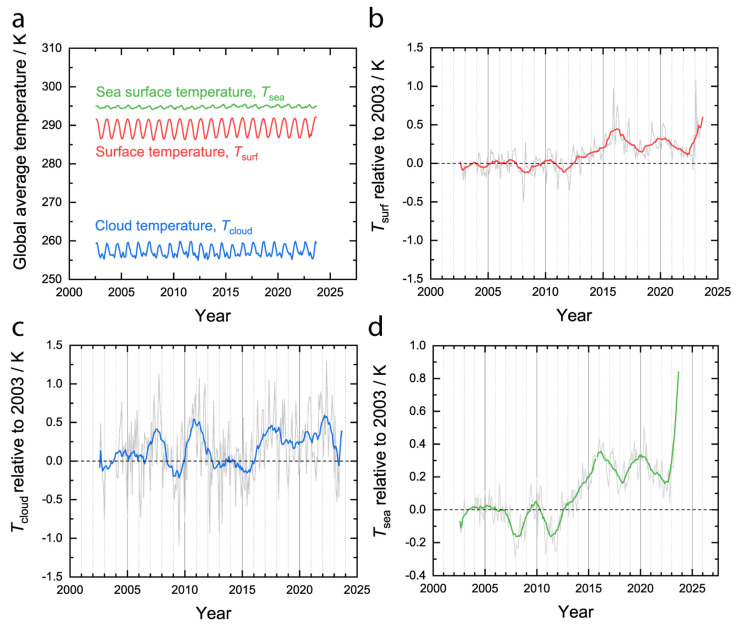
Average surface, cloud, and sea temperatures. (**a**) Area-weighted average surface (red), cloud (blue), and sea (green) temperatures. Area-weighted average (**b**) surface, (**c**) cloud, and (**d**) sea temperatures relative to 2003. Colored lines in (**b**–**d**) are moving averages with a width of 12 months.

Over the same period from 2002 to 2023, the average surface temperature of the Earth has increased but the average cloud temperature has remained approximately constant. Plotted in Figure 5a are the area-weighted average surface, cloud, and sea temperatures without compensating for the annual cycle. The surface temperature is much higher than the cloud temperature, as expected. Plotting the temperatures relative to the same point in the annual cycle of the year 2003 for the surface (Figure 5b), clouds (Figure 5c), and sea (Figure 5d) shows more clearly that the surface has increased in temperature over the last 20 years but the clouds have stayed approximately the same temperature, which is consistent with the greenhouse effect. Moreover, the sea temperature has increased significantly (Figure 5d), and since over 90% of the energy imbalance is accounted for by heating the oceans [30], the rising sea temperature is consistent with positive energy accumulation. While the increased sunlight absorption (Figure 4) played a role in the increasing surface temperature (Figure 5b), it must be noted that the magnitude of the climate forcing due to this decreased albedo is less than the magnitude of the forcing due to the greenhouse effect [23]. Figure 4 and Figure 5 are the crux of the problem for the MEP hypothesis, which has been previously articulated as a criticism [36]. On the one hand, the Earth is absorbing an increasing fraction of the incident sunlight (Figure 4), which should tend to increase the global entropy production rate at constant temperature. On the other hand, the global temperature is increasing (Figure 5), which should tend to decrease the global entropy production rate at constant absorbed sunlight. The outcome of the competition between these two countervailing phenomena for the global entropy production rate is not obvious, and the calculation must be conducted.

**Figure 6:**
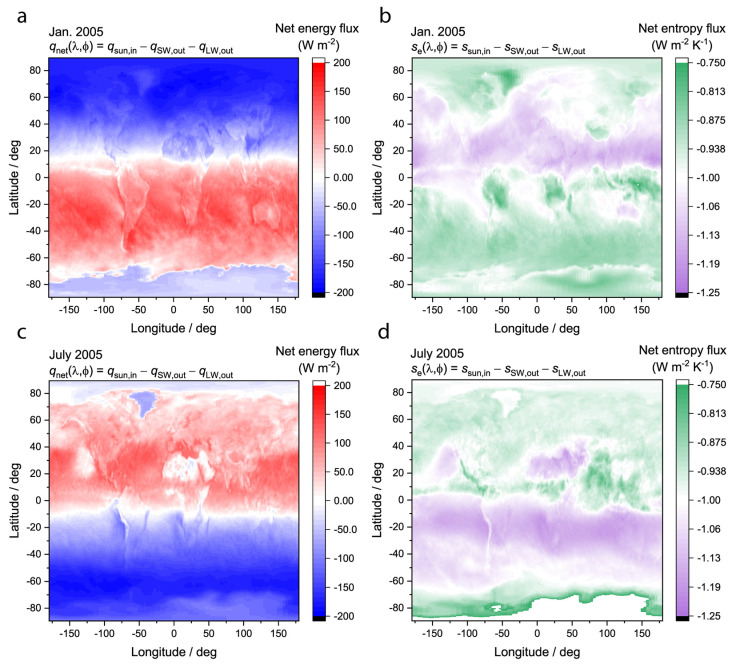
Global distribution of radiation energy and entropy flux during summer and winter. Net energy flux for (**a**) January 2005 and (**c**) July 2005. Net Entropy flux for (**b**) January 2005 and (**d**) July 2005. Note that Antarctica is white in (**d**) because it is greater than −0.75 W m^−2^ K^−1^. Scale was chosen to be the same as (**b**) and emphasize difference between Southern and Northern Hemispheres.

Everywhere in the world, at all times examined in the dataset, the net entropy flux was negative, consistent with earlier results [34], the second law of thermodynamics, and the assumption that radiation comprises the largest energy and entropy exchanges when averaged over a 1° × 1° pixel. The more negative the entropy flux is, the higher the local entropy production rate is due to the radiation exchanges. Plotted in Figure 6a,c are the net energy flux maps for two arbitrary winter/summer months: January 2005 and July 2005. Plotted in Figure 6b,d are the net entropy flux maps for the same months. As expected, the net entropy flux is more negative in the hemisphere experiencing winter because the net energy flux is negative, and the temperature is smaller than the hemisphere experiencing summer. When integrated over the boundary, the portion of the world that receives less solar input is the portion of the world that contributes more to the global entropy production rate. The conclusion is visualized as a coincidence between the blue regions of the net energy flux maps (outgoing net energy flux) and the pink regions of the entropy flux maps (most negative net entropy flux), which change with the season but are correlated at a given time point. Integrating maps such as those in Figure 6b,d over the entire boundary enclosing the Earth using Equations (5)–(7) provides the global entropy production rate in time.

**Figure 7:**
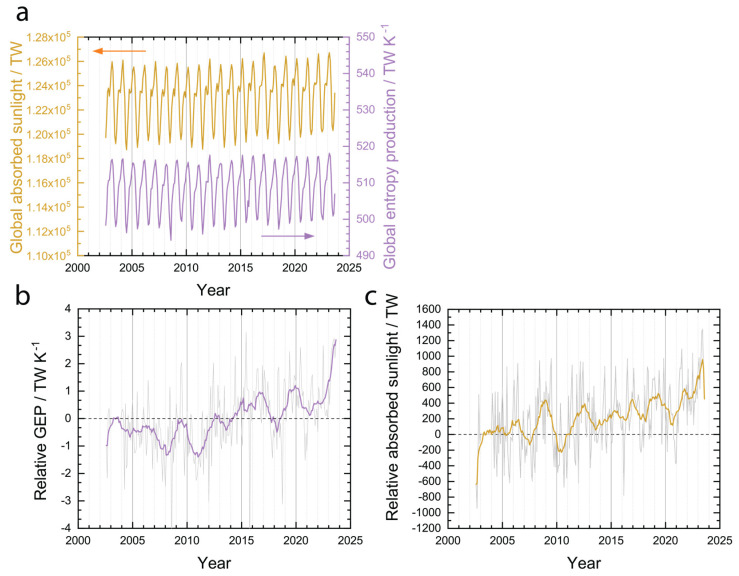
Global absorbed sunlight and entropy production rate. (**a**) Global absorbed sunlight (orange) and entropy production rate (purple). Global entropy production rate (**b**) and absorbed sunlight (**c**) relative to 2003. Colored lines in (**b**,**c**) are moving averages with a 12 month window, grey lines are raw data.

The global entropy production rate has apparently increased over the last 20 years by a magnitude of approximately 1 to 2 TW K^−1^. Plotted in Figure 7a is the global entropy production rate calculated using Equations (5)–(7) without compensating for the annual cycle. The global entropy production rate is highest in the Northern Hemisphere winter, when the Earth is closest to the sun. When the global entropy production rate is plotted relative to the year 2003 to compensate for the annual oscillations, a clear increasing trend can be observed over the last 20 years. As an aside, in an earlier version of this work, a steady state assumption was made for the entropy balance; that is, Equation (7) was set to zero. If the steady state assumption was made, then the global entropy production rate was approximately constant over the last 20 years. The results of the unsteady and steady calculations are consistent with the comments of Gibbins and Haigh that accounting for entropy accumulation in the oceans is necessary to calculate an increasing global entropy production rate [29]. Apparently, the increasing sunlight absorption on Earth over the last 20 years has been enough to compensate for rising temperatures to produce an increasing global entropy production rate.

## 4. Discussion

The present results (Figure 7b,c) are consistent with both the MPP and MEP hypotheses, meaning that neither can be ruled out. The last 20 years have revealed an increased amount of absorbed sunlight (Figure 4b) and an increased global entropy production rate (Figure 7b). Like the simple laboratory example discussed in the introduction, the behavior of the system does not allow discrimination between the hypotheses.

### 4.1. Purpose

To avoid confusion, it is worthwhile to elucidate how the extremum principle, whether it be the MPP or MEP, is envisioned being used. First, it is necessary to outline different types of thermodynamic systems since the extremum principle only applies to certain types of systems. There are the well-known systems that are governed by local equilibrium, which are the subject of numerous engineering thermodynamics textbooks. These systems tend towards a stationary state that is the equilibrium state constrained by the local state variables, for example temperature and pressure. The equilibrium state is subject to an extremum principle-maximum entropy in an isolated system, which can be cleverly defined to model the problem at hand [37]. There are nonequilibrium systems that are maintained by dissipating an external energy flow, external mass flow, or both. Nonequilibrium systems can be either close to local equilibrium, also known as linear nonequilibrium systems, or they can be far from local equilibrium, also known as nonlinear nonequilibrium systems. There are mathematical inequalities that can be used to determine whether a system is either close to or far away from local equilibrium. For a clear discussion, see the lucid book of Prigogine and Kondepudi [38]. Lovelock and Margules succinctly pointed out in their seminal work on the Gaia hypothesis that the Earth operates perpetually with a chemical composition that is far from local equilibrium, meaning that it is in the nonlinear regime. Similar to how entropy maximization is the extremum principle that describes systems governed by local equilibrium, the key idea is that the MPP or MEP act as an extremum principle that governs systems that are constrained to operate far from local equilibrium, such as the surface of the Earth, atmosphere, and the life contained within. The extremum principle is expected to be general to systems in the nonlinear regime of thermodynamics. For example, the author has also applied this idea to describe chemical reactions in a different type of system that operates far from local equilibrium, specifically low-temperature plasma reactors [39,40,41].

Let the term Gaia be used to describe a system with a boundary that contains only the matter on Earth that operates perpetually very far from equilibrium in the nonlinear regime of irreversible thermodynamics, which at a minimum includes all life and human society, but is more expansive and includes information technology, artificial intelligence, and other material objects of the surface and atmosphere. It is tempting to describe increasing the flow of energy through the Earth or increasing the entropy production rate of the Earth, as the purpose of Gaia. However, terms such as purpose, objective, and goal anthropomorphize a system by implying a choice and the ability to act on that choice when no choice may exist. For example, a hot beverage cup, which is governed by local equilibrium, does not have a choice. In the absence of an energy source with a higher temperature, the cup exchanges heat with its environment until the temperatures are equal because that is the natural law it must obey. It is not an anthropomorphic goal, rather Gaia increases the energy flow or entropy production rate because it is governed by a probabilistic natural law described by the MPP or MEP. In other words, systems close to equilibrium are governed by local equilibrium and tend towards equilibrium, while driven nonlinear irreversible systems tend towards maximum energy flow or maximum entropy production rate. The idea is that if the system within the boundary comprising Gaia is to remain as a nonlinear irreversible system, which is required for it to contain life, then it must, subject to its constraints, probabilistically maximize the energy flow through it or its entropy production rate. This all presumes acceptance of MPP or MEP.

### 4.2. Implications for Climate Change Mitigation

At present, neither the MPP or MEP can be ruled out. Thus, the focus will be on global trends that are expected to satisfy both—specifically, increasing the fraction of sunlight that is absorbed by the Earth. If the MEP principle were true, then the analysis is more nuanced because one must also consider temperature and the difficulties with describing it at a global scale. According to the MPP and, arguably, the MEP, societal trends that increase the rate of globally absorbed sunlight are expected to have a higher probability of occurrence than trends that decrease it. Societal trends are defined here to be concerted efforts of large human populations that have significant impacts on the fraction of incident sunlight absorbed by the Earth. The connections between human activity and the absorbed sunlight by the Earth are generally complex and difficult to discern. However, in some instances the connections appear to be relatively clear.

Decreasing albedo is generally expected by MPP and can also be expected from the MEP depending on how surface temperatures change. Thus, activities focused on decreasing albedo are expected to be probable. An interesting approach to decreasing albedo, specifically surface albedo, is to place very large arrays of solar energy harvesting devices such as photovoltaic solar panels in a desert. Since the purpose of these devices is to generate electricity from sunlight, they are typically designed for maximum absorption and have a much lower surface albedo when compared to the naturally occurring surfaces in deserts.

From the vantage point presented in this work, geoengineering strategies [42] appear misguided that aim to lower the surface temperature of the Earth by increasing the albedo, for example using clouds seeded intentionally by aerosols. A concerted effort is unexpected by human society that would cause so much cloud cover that it lowers the surface temperature by reflecting incoming sunlight.

### 4.3. Externalities May Cause Perturbations

There have been periods in the past during which the albedo of the Earth has increased abruptly. For example, in the year 1991, the Pinatubo volcano erupted spewing ash into the atmosphere, causing an immediate increase in albedo [43] and cooling of the atmosphere [44]. Furthermore, prehistoric changes in climate included several transitions from hot-houses to cold-houses [45], including transitions from warm climates to ice ages, and the albedo was presumably higher during those ice ages due to increased snow cover than it was during the preceding warm period. At first glance, these transitions appear to contradict the MPP and MEP, from which decreasing albedo with time is expected. While prehistoric drivers of these climate changes are difficult to know with certainty, evidence suggests that sudden decreases in global average temperature and the commensurate increase in albedo are correlated with the impacts of very large celestial objects on the surface of the Earth, or volcanic eruptions. For example, approximately 66 million years ago, the impact of the bolide that made the Chicxulub crater, which is 150 km in diameter, is believed to have caused the subsequent global average temperature change of approximately −10 °C due to increased albedo from atmospheric aerosol injection [46]. It is remarkable that during the last 540 million years, following excursions to global average temperatures as high as 40 °C and as low as 10 °C, the temperature has repeatedly returned to a relatively stable 20 °C, although it sometimes took 10 million years or more for that stabilization to happen [45]. The observation of damping of the excursions back towards the mean suggests that the causal events originated from outside Gaia, and therefore these events are termed externalities.

Celestial objects obviously originate from outside Gaia and are therefore external to the system that is governed by the MPP or MEP. Since bolides are external to Gaia, they can affect the albedo and radiative temperature in a way that decreases the entropy production rate or absorbed sunlight. Following the impact, provided life is not completely extinguished and the Earth’s surface and atmosphere remain in the nonlinear regime of irreversible thermodynamics, Gaia is expected to begin again towards the extremum, subject to whatever new constraints the externality may have imposed. Thereby the effect of the externality is expected to damp out over long periods of time. In this way, anthropogenic global warming is not a major concern from the perspective of Gaia, although it is a concern for the longevity of humanity.

**Figure 8:**
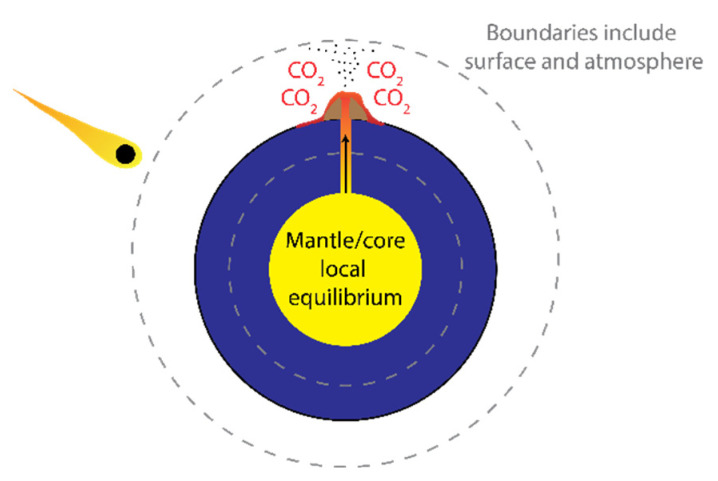
Externalities and revised definition of system boundary. Externalities such as celestial body impacts and volcanic activity are considered boundary interactions and are not part of the system that is subject to MPP or MEP. Note: revision of system boundaries does not change any empirical conclusions of this work (i.e., Figure 2, Figure 3, Figure 4, Figure 5, Figure 6 and Figure 7).

Viewing volcanic impulse inputs, which can also affect albedo and greenhouse gas concentrations, as external to the surface and atmosphere requires redrawing of the boundaries of Figure 1. In Figure 1, the boundary was drawn around the entire Earth, which would include the mantel and core that are the source of volcanic activity. The boundary can be redrawn to include only the atmosphere and surface (Figure 8). The redrawing of the boundary in this way does not affect the energy and entropy balances described above, since the radiation inputs and outputs are so much larger in magnitude than the volcanic activity when averaged over time. However, this redrawing of the boundary makes the volcanic impulses external to Gaia, meaning those inputs are boundary interactions and not subject to the governing principle. They are externalities, and like celestial body impacts, Gaia is expected to damp out the volcanic impulse boundary interactions, which is a notion that is consistent with prehistorical climate evidence [45]. The core and mantel being governed by local equilibrium seems like a reasonable assumption. To our current knowledge, there is no external energy source to supply the core, and the energy flux from the core directed outwards is small with respect to the radiation exchanges with space at the surface.

## 5. Conclusions

In this work, satellite data from the MODIS and CERES instruments were analyzed to determine how the sunlight absorbed by the Earth and entropy generated by the Earth have changed from 2002 to 2023. It was found that the absorbed sunlight has increased significantly over the last 20 years, consistent with recent reports that the Earth albedo has decreased over the same period. The entropy production rate by the Earth appears to have also increased over the last 20 years. Inclusion of entropy accumulation in the oceans in a non-steady entropy balance equation is necessary to see the trend. Uncertainties in the entropy exchanges caused by the temperatures used in the calculations could be reduced if new satellite instruments became available that would directly measure the spectral irradiance of shortwave and longwave outputs with sufficient resolution to calculate the entropy flux as a function of position without the need to specify a temperature. The results presented herein support both the MPP and the MEP. Since evolution towards decreasing albedo can be expected from both principles, societal efforts to harvest sunlight in the desert for electricity production are expected to be successful, but efforts to seed aerosols in the atmosphere to reflect more sunlight are expected to be unsuccessful. Externalities, that is perturbations originating from outside the system comprised of Earth surface and atmosphere, are expected to damp out over geological time periods as global evolution points probabilistically towards the extremum expected by the MPP or MEP. Given that human society has become impactful enough to change the global environment, the results presented herein imply that there may be an intimate connection between the evolution of human society and progress of the system comprised of the Earth surface and atmosphere towards an extremum. That connection may prove useful for forecasting in the social sciences.

## Data Availability

The dataset used for this work is rather large (approximately 3 GB). Specific requests for data should be sent to the author. The CERES and MODIS data products can be downloaded from NASA via the internet.
